# Effect of School District Policy Change on Consumption of Sugar-Sweetened Beverages Among High School Students, Boston, Massachusetts, 2004-2006

**Published:** 2011-06-15

**Authors:** Angie L. Cradock, Anne McHugh, Helen Mont-Ferguson, Linda Grant, Jessica L. Barrett, Steven L. Gortmaker, Claire Wang

**Affiliations:** Department of Society, Human Development and Health, Harvard School of Public Health; Boston Steps, Boston Public Health Commission, Boston, Massachusetts; Food and Nutrition Services, Boston Public Schools, Boston, Massachusetts; Boston Medical Center, Boston, Massachusetts; Department of Society, Human Development and Health, Harvard School of Public Health, Boston, Massachusetts; Department of Society, Human Development and Health, Harvard School of Public Health, Boston, Massachusetts; Department of Health Policy and Management, Columbia Mailman School of Public Health, New York, New York

## Abstract

**Introduction:**

Consumption of sugar-sweetened beverages has increased among youth in recent decades, accounting for approximately 13% of total calories consumed. The Boston Public Schools passed a policy restricting sale of sugar-sweetened beverages in Boston schools in June 2004. The objective of this study was to determine whether high school students' consumption of sugar-sweetened beverages declined after this new policy was implemented.

**Methods:**

We conducted a quasi-experimental evaluation by using data on consumption of sugar-sweetened beverages by public high school students who participated in the Boston Youth Survey during February through April 2004 and February through April 2006 (N = 2,033). We compared the observed change with national trends by using data from the 2003-2004 and 2005-2006 National Health and Nutrition Examination Survey (NHANES). Regression methods were adjusted for student demographics.

**Results:**

On average, Boston's public high school students reported daily consumption of 1.71 servings of sugar-sweetened beverages in 2004 and 1.38 servings in 2006. Regression analyses showed significant declines in consumption of soda (−0.16 servings), other sugar-sweetened beverages (−0.14 servings), and total sugar-sweetened beverages (−0.30 servings) between 2004 and 2006 (*P* < .001 for all). NHANES indicated no significant nationwide change in adolescents' consumption of sugar-sweetened beverages between 2003-2004 and 2005-2006.

**Discussion:**

Data from Boston youth indicated significant reductions in consumption of sugar-sweetened beverages, which coincided with a policy change restricting sale of sugar-sweetened beverages in schools. Nationally, no evidence was found for change in consumption of sugar-sweetened beverages among same-aged youth, indicating that implementing policies that restrict the sale of sugar-sweetened beverages in schools may be a promising strategy to reduce adolescents' intake of unnecessary calories.

## Introduction

Consumption of sugar-sweetened beverages has increased among US children during recent decades (1). Sugar-sweetened beverages are sugary beverages of minimal nutritional value, including soda (ie, carbonated drink containing sweeteners and flavorings) and other sugar-sweetened beverages such as sports drinks, energy drinks, sweetened tea, and fruit drinks. Several studies suggest that excess consumption of sugar-sweetened beverages among youth is associated with poor nutrition and health-related outcomes including increased energy intake and body weight and a lower intake of milk and nutritional components such as calcium (2-4). A potential mechanism that explains the relationship between sugar-sweetened beverages and adiposity is that liquid calories do not produce the same level of satiety as calories in solid forms do. Therefore, calories from food may not be adequately reduced to compensate for the calories consumed via sugar-sweetened beverages, which can result in an excess daily intake of calories (5-7).

Nine of 10 US children and adolescents consume sugar-sweetened beverages or fruit juices on a given day. Per-capita consumption among youth aged 12 to 19 years averages 301 kcal per day or about 13% of their total daily energy intake (1). Although most consumption occurs at home, on a typical weekday 14% to 15% of calories from sugar-sweetened beverages are consumed in school settings (1). Sugar-sweetened beverages sold outside of the National School Lunch Program (ie, competitive foods) are ubiquitous in school settings. Recent national studies suggest that most middle (62%-82%) and high schools (86%-97%) have vending machines (8-10), and more than 90% of secondary schools provide food and beverages à la carte (8). Vending and competitive food sources seldom provide healthful options (8) but are permitted at the discretion of state and local authorities (11). Data from the 2006 School Health Policies and Programs Study suggested that soda or sugar-sweetened fruit drinks (77%) and sports drinks (75%) were available for purchase in most high schools nationwide (10). Furthermore, approximately one-third of high schools reported promoting sales of soda or fruit drinks (36%) or sports drinks (31%) to raise money (10). These school practices can affect the health of students. Among students who use school vending machines, more report buying sugar-sweetened beverages than other beverage types, and school vending machine use is associated with reported overall sugar-sweetened beverage intake (12). According to the 2004 policy statement of the American Academy of Pediatrics, district-wide policies that restrict the sale of sugar-sweetened beverages in schools can safeguard against health problems associated with over-consumption of these beverages (13).

In Boston, a policy that restricts the sale of sugar-sweetened beverages in vending and à la carte settings was approved by the Boston School Committee in June 2004 and initiated with the fall 2004 school year. The new Boston Public Schools Snack and Beverage Policy and subsequent detailed implementation guidelines (14) required that beverages sold in schools or on school grounds adhere to the *Massachusetts à la Carte Food and Beverage Standards to Promote a Healthier School Environment* published by Massachusetts Action for Healthy Kids. The beverage guidelines specifically precluded the sale of soft drinks, fruit drinks (ie, non–100% vegetable or fruit juice beverages), and sports drinks anywhere in school buildings or on school campuses and had specifications that limited other beverage serving sizes. The objective of this study was to determine whether high school students' consumption of sugar-sweetened beverages declined after this new policy was implemented.

## Methods

### Study design

This quasi-experimental evaluation study design (15) contrasted local trends in consumption of sugar-sweetened beverages, measured before and after the policy change was implemented, with national trends in consumption among high-school–aged adolescents. Local data were collected by using cross-sectional data from surveys of Boston public high school students; national data were obtained from the 2003-2004 and 2005-2006 National Health and Nutrition Examination Survey (NHANES).

### Boston public schools and the Boston Youth Survey

The Boston Public Schools system, established in 1647, was the first public school system in the United States. Today it consists of 135 schools, enrolling more than 55,000 students (fiscal year 2010). The student body is diverse: 37% of students are black, 39% are Hispanic, 13% are white, and 9% are Asian, and approximately 74% of students are eligible for free or reduced-price meals. The 2009-2010 school year budget funded 9,023 staff positions including more than 4,600 teachers, most of whom (98%) are licensed in their teaching assignment (16).

In February through April 2004 and again in 2006, the City of Boston and the Harvard Youth Violence Prevention Center conducted the Boston Youth Survey among students in grades 9 through 12 in Boston's public high schools. Trained survey administrators collected data using a 2-level sampling method modeled after the Youth Risk Behavior Surveillance System; classrooms within schools selected from among the total number of eligible public high schools in Boston were sampled. Researchers repeated this sampling procedure in 2006 to obtain data from 2 cross-sectional student samples. Students with complete data on demographic covariates and consumption of sugar-sweetened beverages were included in this analysis. In 2004, 1,079 students from 17 high schools participated in the survey (n = 1,079), and in 2006, students from 18 high schools participated (n = 1,233). Students anonymously completed surveys during specified class periods, and the surveys covered topics including education, mental health, nutrition and physical activity, and violence-related issues (17). The data were not weighted but represent findings from the final sample of students for the given year. The Office of Human Research Administration at the Harvard School of Public Health approved the study protocol.

### Measures

Students answered 2 questions to assess total consumption of sugar-sweetened beverages. The questions asked, "In the past seven (7) days, how often did you drink soda (not diet)?" and "In the past seven (7) days, how often did you drink Hawaiian Punch, lemonade, Kool-Aid or other sweetened fruit drinks?" A serving was further defined as 1 "can" or "glass." Respondents were instructed to count a 20-oz bottle as 2 cans. Seven response option categories ranged from "never or less than 1 can per week" to "3 or more cans per day." For analysis, researchers used the midpoint of each reported category (eg, 1-2 cans/d was recoded to 1.5 servings/d). The measure of total sugar-sweetened beverage servings per day included reported consumption of soda and other sugar-sweetened beverages (2). Student demographic characteristics including age, grade, race/ethnicity, and primary neighborhood of residence (zip code and neighborhood name) were obtained via survey questions. Response categories for self-identified race/ethnicity varied slightly in 2004 and 2006 survey questions. To ensure consistency, we collapsed available response options in each year into larger categories for analysis (ie, white [non-Hispanic]; black or African, Cape Verdean, or Caribbean [non-Hispanic]; Hispanic or Latino; Asian or Pacific Islander [non-Hispanic]; and other race or ethnicity [including multiracial]). Complete data were available for 895 (83%) of 1,079 students in 2004 and 1,138 (92%) of 1,233 students in 2006. In 2004 and 2006, respectively, 3.5% and 2.8% of respondents were missing data on consumption of sugar-sweetened beverages.

### Analysis

We used linear regression analysis to examine changes in mean servings per day of sugar-sweetened beverages between 2004 and 2006, adjusting for potential differences in student composition. In regression models, we estimated change in consumption via an indicator variable identifying surveys completed in 2006 (postpolicy change; 2004 survey was reference), controlling for respondents' sex, grade, race/ethnicity, and primary neighborhood of residence. The coefficient estimate from these models provided an estimate of the effect of the policy change on average daily consumption between 2004 and 2006 among those students participating in the Boston Youth Survey, controlling for changes in demographic composition over time. Significance was set at *P* < .05, and all analyses were conducted by using SAS version 9.1 (SAS Institute, Inc, Cary, North Carolina).

### National trend comparison: NHANES

We obtained dietary recall data from adolescents aged 15 to 19 years surveyed during the NHANES 2003-2004 and 2005-2006 periods. NHANES is an ongoing national survey conducted by the National Center for Health Statistics (NCHS) using a multistate, clustered, probability sampling strategy that provides nationally representative estimates of selected health outcomes and health-related behaviors. Complete details on the data collection procedures and guidelines for analysis of data are available on the NCHS website (18).

For analysis of national trends in consumption of sugar-sweetened beverages, we used the 24-hour recall interview component of the NHANES survey that documented the type, quantity, and location of each beverage consumed. We included data from the 2003-2004 (N = 1,196) and 2005-2006 (N = 1,233) samples of adolescents who completed a single 24-hour dietary recall. The methods for quantifying consumption via NHANES data have been detailed in previous studies (1). Briefly, researchers coded beverage items according to the United States Department of Agriculture (USDA) Food and Nutrient Database, a source that provides nutritional content on the basis of standardized recipes, and calculated the total calories derived from each beverage consumption occasion as indicated by the self-reported consumption quantity and the unit caloric content provided by the USDA database. The outcome measure of per capita total kilocalories consumed per day from sugar-sweetened beverages included consumption of soda, sport drinks, fruit drinks and punches, low-calorie drinks, sweetened tea, and other sweetened beverages. All analyses were weighted to be representative of the US population and to account for the complex sampling design of NHANES. Regression analysis including covariates for age, sex, and race/ethnicity (non-Hispanic white, non-Hispanic black, and Mexican American) accounted for potential differences in the sample population demographics across the 2 survey periods. We excluded from analysis data from respondents who self-identified as "other" race/ethnicity because of the small sample size.

### Differences in measurement strategies

The Boston Youth Survey and the NHANES recall have several methodologic differences. The Boston Youth Survey has a 7-day recall horizon, and the NHANES recall covers the previous 24-hour period. Most NHANES recall responses began on a weekday (58%). Eighty-seven percent of adolescents' observations were obtained during the school year but not during vacation periods. The Boston Youth Survey is self-administered as a pencil-and-paper survey, and NHANES uses an interviewer-administered survey. The Boston Youth Survey asks students to report consumption of sugar-sweetened beverages in 2 categories, and the NHANES recall does not use predefined categories of sugar-sweetened beverages but rather asks about all beverages consumed during the previous 24 hours (from 12:01 am to midnight), recording both the type and amount consumed. The NHANES recall uses a national database to define ingredient and nutrient content of beverages consumed, and we calculated the measure of sugar-sweetened beverage consumed using methods established in previous research (1).

## Results

### Boston

High school students participating in the 2004 and 2006 Boston Youth Survey were of generally comparable demographic composition (Table 1). Slightly more than half of the students were female (55% in 2004, 57% in 2006). The sample in 2006 was slightly older: approximately 15% of the sample was in grade 12 versus 7% in 2004. Question wording assessing student racial/ethnic background was not identical in the 2004 and 2006 surveys. However, the distribution of the study sample across categories of race/ethnicity was similar in 2004 and 2006. Students from all Boston neighborhoods participated in the survey.

In 2004, students reported an average daily consumption of 0.81 servings of soda and 0.90 servings of other sugar-sweetened beverages, for a total of 1.71 servings per day. Average daily consumption of all beverages declined from 2004 to 2006 (Table 1). The decline in mean consumption from 2004 to 2006 was significant for soda (−0.16 servings/d), other sugar-sweetened beverages (−0.14 servings/d), and total sugar-sweetened beverages (−0.30 servings/d) (Table 2). The proportion of students reporting no sugar-sweetened beverage consumption during the  week preceding the survey more than doubled from 4.5% in 2004 to 9.8% in 2006.

### United States

In 2003-2004, adolescents aged 15 to 19 years reported a mean per capita daily consumption of sugar-sweetened beverages of 339 calories, corresponding to 1.74 servings per day (Table 3). In 2005-2006, adolescents reported consuming an average of 331 calories per day in sugar-sweetened beverages, or 1.66 servings. In a regression model adjusting for age, sex, and race/ethnicity of survey respondents, we found a small but nonsignificant decline in daily per capita consumption of sugar-sweetened beverages nationally between 2003-2004 and 2005-2006 (−0.08 servings/day; 95% CI −0.27, 0.11; *P* = .41), indicating no significant change in consumption of sugar-sweetened beverages nationally between 2003-2004 and 2005-2006.

## Discussion

Previously reported national data have indicated an upward trend in consumption of sugar-sweetened beverages among children and adolescents between 1988 and 2004 (1). However, after the implementation of a policy change in the Boston Public Schools restricting the sale of sugar-sweetened beverages on school grounds, we found a reduction in consumption among Boston students, although national data suggest no change in consumption among high-school aged youth during the same period. The magnitude of the decline in consumption of sugar-sweetened beverages after the policy change in Boston Public Schools corresponds to approximately 45 kcals per day, assuming there are 150 kcals per 12-oz serving (www.pepsiproductfacts.com). This estimate is commensurate with national estimates of caloric consumption of sugar-sweetened beverages consumed by youth during school (approximately 42.5 kcals/d [1]), suggesting that the observed decline in caloric intake could plausibly be accounted for by the elimination of opportunity for consumption of sugar-sweetened beverages during school. Boston's results also suggest that youth may not compensate for in-school restrictions on sugar-sweetened beverages by increasing consumption outside of school.

Reductions in consumption of sugar-sweetened beverages, if maintained over time and not compensated for by an increase in other dietary intake, could have substantial health impact. A 45 kcal reduction in daily energy consumption could eliminate 25% to 40% of the total excess calories, or "energy gap" (110 kcals-165 kcals/day), that is attributed to increasing average body weight among US children (19). National data that document changes in drinking patterns suggest that replacing all sugar-sweetened beverages with water among youth could result in an average reduction in total energy intake of 235 kcals/day (20), and 1 study has documented a significant reduction in obesity among elementary school students after an intervention provided water fountains in schools (21).

Boston's success highlights the importance of implementing comprehensive policies and strategies to restrict the sale of sugar-sweetened beverages in all school settings (11) in conjunction with promoting reductions in consumption of sugary drinks among young people. The passage of the Boston Public Schools beverage policy was the beginning of more widespread focus on promoting more healthful foods and beverages in Boston and Boston schools. For example, further initiatives included the implementation of nutrition-related curricula in middle and primary schools and interdepartmental committees and collaborations charged with monitoring implementation and acceptance of related policy guidelines. Awareness-raising activities in the Boston Public Schools system included a presentation of the new policy guidelines to principals before implementation, parent workshops on healthful snack choices, dissemination of pamphlets to teachers and school staff detailing alternatives for fundraising, and a brochure for school administrators and teachers entitled *Healthy Beverages and Snack News*. Boston city officials also negotiated new procurement contracts with vendors who would supply the new more healthful options to schools, and school vending machines were stocked with water and 100% juice instead of sugar-sweetened beverages.

Nationally, the sales to schools of beverage products that meet nutrition standards are tracked as part of the 2006 memorandum of understanding (MOU) signed by the Alliance for a Healthier Generation, the American Beverage Association, and 3 major beverage producers (22). Data from 2004 to 2006-2007 indicate a decline in overall shipment quantities, particularly in full-calorie carbonated drinks (ie, soda), and increases in the proportion of products shipped that were water or other nonsoda drinks that met the MOU nutritional standards (22). These data also suggest that if schools were to eliminate all sugar-sweetened beverages, the per-student consumption would drop by about 3.5 oz per day, an amount similar to that found in Boston (3.8 oz).

However, although the increased availability of more healthful beverage options in schools is heartening, strong policies and continued implementation and monitoring are still needed. A recent update on beverage shipments by bottlers suggests that in US high schools alone in 2008-2009, 1.2 billion ounces of full-calorie carbonated soft drinks (18.9% of the product mix) and 1.3 billion ounces of sports drinks (19.8% of the product mix) were still available to students (23). Given the large quantities of sugar-sweetened beverages still available in school settings, continued policy and environmental efforts at the community, state, and national levels may be necessary to promote and encourage more healthful beverage choices for our nation's young people.

Several limitations of this study merit discussion. We analyzed data from a single community. Nutrition education and health promotion activities focused on consumption of sugar-sweetened beverages separate from the policy change in Boston may play a role in the observed decline in overall consumption of sugar-sweetened beverages. Potentially, those community and school-based education and awareness-raising activities in Boston may have increased student knowledge about consumption of sugar-sweetened beverages, thus contributing to the observed effects that we associate with the policy change. However, school-based education and awareness activities can be considered part of a comprehensive policy implementation strategy, contributing to both policy adherence and compliance within schools. Furthermore, NHANES and Boston Youth Survey estimates of consumption of sugar-sweetened beverages are not directly comparable because of differences in wording and data collection methods. Additionally, accessible alternatives for water in Boston may not be comparable to those in other communities. Although bottled water was available in all Boston schools, a lack of accessible water fountains may have served to increase demand for less-healthful beverages. According to data collected during the 2006-2007 school years, only 14% of Boston Public Schools provided access to public drinking water via water fountains or bubblers (unpublished data, Boston Public Health Commission, obtained October 20, 2008.)

Data from Boston youth indicate that significant reductions in sugar-sweetened beverage intake coincided with a policy change that restricted the sale of sugar-sweetened beverages in public high schools. Because no national evidence has been found for change in consumption of sugar-sweetened beverages among same-aged youth, such policy changes may be promising strategies to reduce unnecessary caloric intake.

## Figures and Tables

**Table 1:** Characteristics of Boston High School Students Participating in the Boston Youth Survey, 2004 and 2006

**Characteristics**	2004 (N = 895)^a^	2006 (N = 1,138)^a^
**Demographic, n (%)**		
**Sex**		
Female	491 (55)	650 (57)
Male	404 (45)	488 (43)
**Grade**		
9	355 (40)	335 (29)
10	258 (29)	298 (26)
11	223 (25)	332 (29)
12	59 (7)	173 (15)
**Race/ethnicity^b^**		
Non-Hispanic white	99 (11)	143 (13)
Black or African/Cape Verdean/Caribbean	384 (43)	516 (45)
Hispanic/Latino	276 (31)	335 (29)
Asian/Pacific Islander	78 (9)	71 (6)
Other/multiracial	58 (6)	73 (6)
**Primary neighborhood of residence**		
Allston/Brighton	38 (4)	52 (5)
Combined Central^c^	55 (6)	51 (4)
Charlestown	17 (2)	27 (2)
Dorchester	336 (37)	437 (38)
East Boston	81 (9)	28 (2)
Hyde Park	57 (6)	122 (11)
Jamaica Plain	41 (5)	59 (5)
Mattapan	61 (7)	86 (8)
Roslindale	67 (8)	98 (9)
Roxbury	91 (10)	102 (9)
South Boston	34 (4)	43 (4)
West Roxbury	17 (2)	33 (3)
**Beverage consumption, mean (95% CI)**		
Servings/d of SSBs^d^	1.71 (1.61-1.81)	1.38 (1.30-1.47)
Servings/d of soda	0.81 (0.74-0.87)	0.63 (0.58-0.67)
Servings/d of other SSBs	0.90 (0.84-0.97)	0.76 (0.70-0.81)

Abbreviations: CI, confidence interval; SSBs, sugar-sweetened beverages.

a Sample includes students with complete data on demographic covariates and SSB consumption, representing 83% and 92% of all students surveyed in 2004 and 2006, respectively.

b Race and ethnicity questions were not identical in 2004 and 2006 Boston Youth Surveys and are not directly comparable. Response options were collapsed into these larger categories for each year.

c Some neighborhood data were collapsed because of small sample sizes.

d SSBs included soda and other sugar-sweetened drinks such as fruit punch and lemonade. One serving was defined as 1 "can" or a similar 12-oz serving. Respondents were instructed to count a 20-oz bottle as 2 cans.

**Table 2:** Change^a^ in Daily Servings of Sugar-Sweetened Beverages (SSBs) Consumed Among Boston High School Students, Boston Youth Survey, 2004-2006

Type of Beverage	Crude Model^b^			Model Adjusted for Covariates^c^		

	Estimate (95% CI)	*P* Value^d^	*R* ^2^	Estimate (95% CI)	*P* Value^d^	Adjusted *R* ^2^
Total SSBs^e^	−0.32 (−0.45 to −0.19)	<.001	0.01	−0.30 (−0.43 to −0.17)	<.001	0.06
Soda	−0.18 (−0.26 to −0.10)	<.001	0.01	−0.16 (−0.23 to −0.08)	<.001	0.03
Other SSBs	−0.14 (−0.23 to −0.06)	<.001	0.01	−0.14 (−0.23 to −0.06)	<.001	0.06

Abbreviations: Estimate, coefficient estimate for change; CI, confidence interval.

a Change from baseline survey year (2004) to follow-up year (2006) after policy change that restricted the sale of SSBs on school grounds was implemented.

b Crude models include indicator for data collection after policy change only.

c Covariates in adjusted models are sex, grade, race/ethnicity, and primary neighborhood of residence.

d *P* value obtained from *t *test for coefficient estimated using multiple linear regression.

e SSBs included soda and other sugar-sweetened drinks such as fruit punch and lemonade. One serving was defined as 1 "can" or a similar 12-oz serving. Respondents were instructed to count a 20-oz bottle as 2 cans.

**Table 3:** Characteristics of Adolescents Participating in the National Health and Nutrition Examination Survey, 2003-2004 and 2005-2006

**Characteristics**	2003-2004 (N = 1,196)^a^	2005-2006 (N = 1,233)^a^
**Demographic, n (%^b^)**		
**Sex**		
Female	540 (47)	618 (47)
Male	656 (53)	615 (53)
**Age, y**		
15	228 (20)	250 (23)
16	252 (20)	260 (21)
17	255 (21)	234 (18)
18	234 (21)	265 (21)
19	227 (18)	224 (17)
**Race/ethnicity**		
Non-Hispanic white	363 (72)	358 (71)
Non-Hispanic black	455 (16)	456 (17)
Mexican American	378 (12)	419 (12)
**Beverage consumption, mean (95% CI)**		
kcals/day from SSBs consumed	339 (311-367)	331 (278-385)
Servings/day of SSBs	1.74 (1.64-1.83)	1.66 (1.47-1.85)

Abbreviations: CI, confidence interval; SSBs, sugar-sweetened beverages.

a The samples for this analysis were restricted to adolescents reporting non-Hispanic white, non-Hispanic black, or Mexican American race/ethnicity only.

b Percentage of US population estimated with weights to adjust for unequal probability sampling.
